# Delivery of Niacinamide to the Skin Using Microneedle-Like Particles

**DOI:** 10.3390/pharmaceutics11070326

**Published:** 2019-07-11

**Authors:** Chong In Shin, MunSik Kim, Yeu-Chun Kim

**Affiliations:** Department of Chemical and Biomolecular Engineering, Korea Advanced Institute of Science and Technology (KAIST), 291, Daehak-ro, Yuseong-gu, Daejeon 34141, Korea

**Keywords:** transdermal drug delivery, niacinamide, microneedle, microparticle, cosmeceutical

## Abstract

The stratum corneum is the outermost skin layer that obstructs the delivery of active ingredients found in cosmeceutical products. Chemical peels and microbeads have been used to overcome this layer, but these methods can cause side effects and are not environmentally friendly. While microneedles do not share the dangers mentioned above, they are currently only available as patches, which makes them unsuitable to be used with products that are usually applied onto a large area of the skin surface. Therefore, the aim of this study was to develop microneedle-like particles (MLP) whose needles would disrupt the skin during the rubbing process. A modified approach taken from conventional micromolding techniques was used to make the MLPs. The experimental results show that the fabricated structures had the required mechanical strength. Furthermore, after the application of the MLPs, the permeability of two fluorescent dyes, fluorescein sodium salt and sulforhodamine B increased to 217.6% ± 25.6% and 251.7% ± 12.8% respectively. Additionally, the permeability of a model drug, niacinamide, was shown to have increased to 193.8% ± 29.9%. Cryosectioned porcine slices also confirmed the ability of MLPs to enhance skin permeability by revealing a deeper penetration of the applied fluorescent dye. Altogether, the results demonstrate the potential of MLPs to be used as safe skin permeability enhancers that can be applied all over the skin.

## 1. Introduction

The skin layer consists of the epidermis, dermis and hypodermis, but it is the outermost layer of the epidermis called the stratum corneum (SC) that has a critical role in protecting the body from the outside environment [1]. The 10–20 μm thick SC has a brick and mortar structure in which corneocytes act as bricks, and intercellular lipids act as the mortar [2]. This lipid structure prevents water loss through the epidermis, but at the same time, it prevents foreign substances, especially hydrophilic ones, from entering the skin [3]. As a result, numerous methods such as chemical peels and microbeads have been developed to overcome this barrier [4,5]. However, the aforementioned strategies can cause side effects and are harmful to the environment [6].

Microneedles, on the other hand, are available in various forms and are capable of disrupting the SC to enable both small and large molecules to permeate the skin [7,8,9,10]. Additionally, microneedles are painless because the needles are short enough that they do not reach the nerve fibers residing in the dermis [11]. Due to these attributes, microneedles have been used to deliver numerous substances such as vaccines, therapeutic agents, and cosmeceutical agents [12,13]. Even though the skin is the largest organ of the body, accounting for about 15% of the total body weight in adult humans, currently, microneedles are only available in patches, and the area of the skin that these patches can cover is limited [14].

To the best of our knowledge, particles that incorporate microneedle-like features and are able to disperse across the skin have never been developed before. Here, we report for the first time, microneedle-like particles (MLP) made of fast dissolving biocompatible polymers that combine the disrupting ability of microneedles and the large coverage area of microbeads without leaving any waste. MLPs are micrometer sized particles that have a flat bottom and contain microneedles that protrude in an x,y,z direction, making them part of a 2.5 dimensional structure. The PVA/sucrose matrix of the needle makes it environmental friendly, while at the same time, the 2.5-dimensional structure of the needle enables it to be applied onto a large skin area to enable better skin permeability of the widely used active ingredients in the cosmeceutical industry (Table 1). Traditionally, active ingredients such as niacinamide have been applied topically or after physically disrupting the skin via dermabrasion. Delivery via topical application is hindered by the skin layer, while a separate instrument and a trained medical professional is required in order to perform dermabrasion. MLPs, however, can easily be applied onto the skin without any additional equipment and enhance the delivery of niacinamide by just rubbing it onto the skin. Hence, the aim of this study was to determine the effectiveness of the newly designed MLPs to disrupt the skin layer and enhance the permeability of different active ingredients.

According to Ryan F. Donnelly’s research, they treated Candida albicans, Pseudomonas aeruginosa, and Staphylococcus epidermidis culture media on a Silescole, which is a stratum corneum mimic, to determine the risk of hypodermic needle and microneedle infection. After 24 h, the total number of microorganisms in the Franz cell’s receptor fluid was counted. None of the microneedle treated groups were found. But, in the hypodermic needle group, C. albicans (48.0%) and S. epidermidis (3.5%) were passed [15]. In other words, it can be seen that the microneedle is more safe injection method than existing injection methods. And according to Mark Prausnitz’s paper, there are many factors related to the risk of infection due to microneedle, but it is expected that there will be no serious safety problems in the clinical phase. This is because skin barriers are periodically breached and regenerated, and small abrasions are common, but infections are very rare [16]. Thus, drug delivery using MLPs also appears to have a low risk of infection.

## 2. Materials and Methods

### 2.1. Materials

Poly(vinyl alcohol) (PVA, Mw 13,000–23,000, 87–89% hydrolyzed), sucrose, sulforhodamine B (SRB), poly(methyl methacrylate) (PMMA), fluorescein sodium salt (FSS), and niacinamide were purchased from Sigma-Aldrich (St. Louis, MO, USA). Ethyl Acetate was purchased from Daejung Chemicals and Metals (Daejeon, Korea). Hard-polydimethylsiloxane was purchased from Dow Corning (h-PDMS, MS-10021, Dow Corning, Midland, MI, USA). Porcine skin was purchased from Cronex (Cheongju, Korea). Milli-Q (Millipore, Burlington, MA, USA) water was used for all the experiments.

### 2.2. Fabrication of Mold

The MLP was designed using Solidworks (Dassault Systemes, Mason, OH, USA) to generate the stereolithography (STL) file required for the fabrication of the positive mold. A block of steel was polished and electro-discharge machined according to the design to produce the positive master mold, which was then repeatedly used to produce the negative molds made of h-PDMS. The h-PDMS silicone base was mixed with its curing agent at a ratio of 1:1, and the bubbles that formed during mixing were removed by centrifugation at 3000 rpm for 1 min (Hanil Science Medical Supra 22K, Daejeon, Korea). The silicone mixture was poured onto the steel mold and left to bake at 80 °C for 4 h in an oven.

### 2.3. Fabrication of the MLPs

PVA (15 *w*/*v*%) and sucrose (15 *w*/*v*%) were dissolved in distilled water. 100 μL of the mixture were pipetted onto the h-PDMS molds and vacuumed in a desiccator for 4 min. Then, the excess layer mixture was carefully removed, and 100 μL of a fresh layer of the same mixture was applied and left to air-dry in a desiccator for 24 h. After drying, the added layer was carefully removed, and 100 μL of PMMA solution (7% *w*/*v*) dissolved in ethyl acetate were applied and left to dry in a hood. The MLPs were removed from the mold along with the PMMA film and submerged in ethyl acetate solution to dissolve the film and free the MLPs. Finally, the MLPs were washed twice and left to dry in a hood. After drying, the MLPs were examined using a stereo microscope (Leica S6 D, Leica Microsystems Ltd., Wetzlar, Germany).

### 2.4. Mechanical Test

To measure the mechanical strength, a 2 × 2 array of MLPs was placed on a precision balance, and a metal bar slowly traveled downward against the array at a speed of 10 μm/s. The compression of the MLPs was visually recorded, and the applied force and displacement were plotted on a graph.

### 2.5. Franz Cell Test

To assess the ability of the MLPs to enhance transdermal drug delivery, SRB and FSS and niacinamide were used as model drugs. The individual MLPs released from the PMMA baseplate were laid on top of the porcine skin with an exposed circular area of 0.8 cm^2^ and hand-rubbed in a circular motion. The effect of the number of MLPs was observed by applying 600 and 1200 MLPs for 30 s while the effect of the application time was also observed by applying 1200 MLPs for 30 and 60 s. The penetration of the model drug was analyzed using Franz diffusion cells (FCDV-15, Lab Fine, Inc., Anyang, Korea) with a diffusion area of 0.785 cm^2^ and a receptor compartment of 5 mL. After treating the skin with the MLPs, it was mounted between the donor and receptor chamber of the diffusion cells and secured with a clamp. The receptor chamber was filled with PBS and allowed to equilibrate at 37 °C while stirring at 200 rpm. The donor chamber was filled with 3 mL of 1 mg/mL PBS solution of the model drug. After 3, 5, and 24 h, sample volumes from the receptor chamber were extracted and replaced with fresh PBS of the same volume. The amount of drug permeated through the skin was analyzed using HPLC (YL9100 HPLC System, YL Instruments, Anyang, Korea) and a spectrophotometer (SpectraMax Gemini XPS, Molecular Devices, San Jose, CA, USA) and expressed as the percentage relative to the control.

### 2.6. Histological Analysis

After treating porcine skin with MLPs, SRB (1 mg/mL) solution was applied for 10 min, at room temperature, and the skin samples were then frozen overnight in a deep freezer. The frozen skin samples were embedded in optimum cutting temperature (OCT) compound and solidified in an isopentane solution with dry ice for fixation and sectioned into 30 μm thick slices using a cryostat (CM1850, Leica Microsystems Ltd., Wetzlar, Germany). The sectioned slices were mounted onto glass slides and examined using a fluorescence microscope (DM2500, Leica Microsystems Ltd., Germany).

## 3. Results and Discussion

### 3.1. Fabrication of the Mold

**Figure 1:**
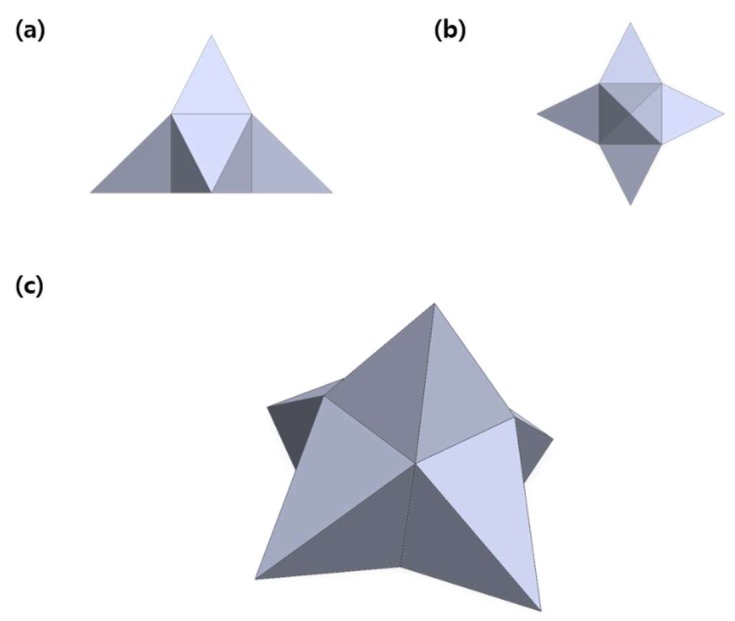
3D Computer-aided design (CAD) images of the designed MLP displaying the (**a**) side view, (**b**) top view, (**c**) and overall view. Each MLP had a height of 300 μm, a base length of 450 μm, a needle length of 150 μm, and a tip diameter of 30 μm.

The MLP structure was designed with its application in mind, and thus, it was necessary that the needles would be able to disrupt the skin while being applied omnidirectionally. Originally, a design that was fully three dimensional was conceived, but mass producing such a complex structure would have been impossible using already existing micromolding methods. Therefore, a new design with five needles in the x, y, and z direction were incorporated into the structure to enable the disruption of the skin. Furthermore, instead of a full three dimensional structure, the bottom of the MLP was made flat to mass produce the particles using conventional micromolding techniques shown in Figure 1. Due to the complex structure of the MLPs, typical mold fabricating techniques such as lithography could not be used to make the master mold, and instead, electro discharge machining (EDM) process was applied to produce steel MLPs [17]. The resulting array contained 121 MLPs (11 × 11) in an area of 1 cm^2^, and each MLP had a height of 300 μm, a base length of 450 μm, a needle length of 150 μm, and a tip diameter of 30 μm.

### 3.2. Fabrication of the MLP

**Figure 2:**
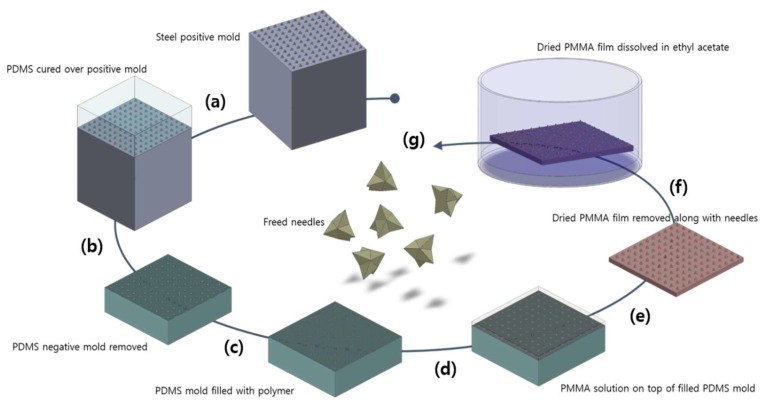
Fabrication process of the MLPs. (**a**) h-PDMS is poured onto the positive steel mold. (**b**) The cured h-PDMS negative mold is removed. (**c**) The negative h-polymethylsiloxane (PDMS) mold is filled with a polymer mixture via vacuum and then dried. (**d**) The PMMA solution in the ethyl acetate is spread on top of the h-PDMS mold and left to evaporate. (**e**) The dried PMMA film is removed from the mold and peeled off along with the dried MLPs. (**f**) The PMMA film is submerged in ethyl acetate for dissolution. (**g**) Needles are freed from the PMMA film and recovered from the ethyl acetate. Images created using SolidWorks (SolidWorks 2015, Dassault Systèmes, Vélizy-Villacoublay, France).

The MLPs were fabricated with a slight modification of conventional micromolding techniques used to produce microneedle patches [18]. First, h-PDMS was poured onto the positive steel mold and cured until it became solid (Figure 2a). The produced h-PDMS negative mold was removed from the positive steel mold and was filled with a PVA/sucrose mixture solution using a vacuum. Non-dissolving substances like silicone are not FDA approved, and broken silicone or metal microneedles have been shown to cause skin problems [19]. However, PVA and sucrose already have established safety profiles and clinical applications which minimize safety concerns regarding their use (Figure 2b,c) [20]. Next, the excess polymer solution was carefully removed and recycled for future use. Due to the unique character of the MLPs, it was essential to completely remove the excess from the mold surface, leaving the polymer solution only in the mold cavities. However, this was not possible using normal PDMS because its low hardness would cause the polymer in the mold cavity to be removed during the excess removal process. Therefore a harder type of PDMS called h-PDMS was used to leave the polymer solution in the mold cavity intact. Additionally, after the drying process, the polymer was observed to shrink and create a cavity at the center of the MLP structure. It has been reported that the mixture concentration and viscosity, back-film thickness, and drying temperatures all affect the depth of the cavity formed [21,22]. Using a PVA/sucrose mixture formulation of 30 *w*/*v*% and adding 100 μL of this mixture onto the h-PDMS mold was shown to minimize the formation of cavity. Once the polymer dried completely, it was essential to remove the individual MLPs altogether. One study demonstrated the fabrication of microneedles using PVA molds in which the mold was filled with PMMA and then dissolved in water producing PMMA microneedles [23]. This concept was used to cast a PMMA solution in ethyl acetate over the mold and left to evaporate to form a PMMA film. (Figure 2d). This film served as a backing for the MLPs inside the cavity of the molds, and it was carefully peeled off along with the needles (Figure 2e). Using PMMA and ethyl acetate was essential during the fabrication process because the PMMA is soluble in ethyl acetate while the PVA/sucrose mixture is insoluble. This difference in solubility enabled the dissolution of the PMMA film in ethyl acetate and the release of the MLPs without affecting their structure (Figure 2f). Finally, the needles were collected and used for further experiments (Figure 2g).

**Figure 3:**
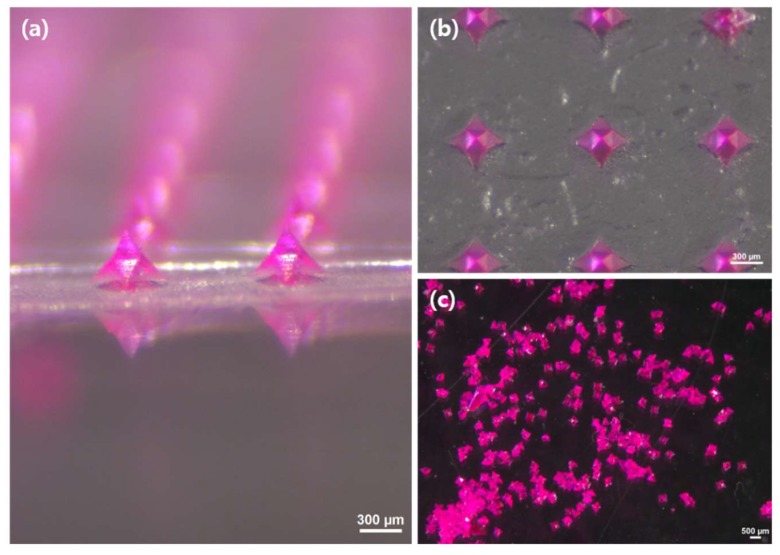
MLP array loaded with SRB (**a**) observed by stereo microscopy. The array contained 121 MLPs (11 × 11) in an area of 1 cm^2^. The MLPs were (**b**) removed from the h-PDMS mold by peeling off the PMMA film. The film was then (**c**) dissolved in ethyl acetate, and the freed MLPs were collected.

By following the modified micromolding method, individual MLPs were successfully created. To visualize the needles, SRB was mixed onto the MLP matrix solution. As shown in Figure 3a,b, the dye loaded MLPs were clearly removed from h-PDMS mold with high accuracy along with the transparent dried PMMA film. Figure 3c shows the released and intact needles once the PMMA backing was dissolved in ethyl acetate. This approach enabled the easy fabrication of the MLPs and was suitable to make hundreds of particles at once.

### 3.3. Mechanical Test

**Figure 4:**
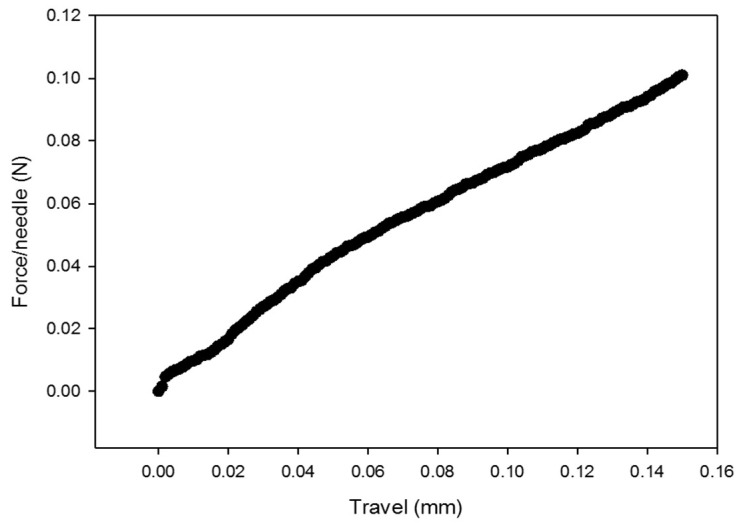
Force travel-curve of the MLPs.

For the MLPs to properly disrupt the skin during their application, it is necessary that the tip of the needles do not break and that the overall structure has the required mechanical strength to withstand the force applied during rubbing. A vertical metal rod slowly moved towards a 4 × 4 array of MLPs placed on a precision balance, and the force recorded was converted into the force applied per needle. As shown in Figure 4, the compression force steadily increased from 0 to 0.1 N when the MLPs were compressed from 0 mm to 0.15 mm. If the microneedles are not designed or fabricated with the proper mechanical strength, they are prone to failure during application which can be observed by a discontinuous point in the force-travel curve [24]. However, the force-travel curve of the MLPs showed no sudden decrease in force meaning that no bending or fracture of the needles took place, which made them strong enough to be rubbed against the skin.

### 3.4. Transdermal Delivery of Fluorescent Dyes after the MLP Treatment

To ensure that the fabricated MLPs were able to disrupt the skin and hence increase the skin permeability of drugs, two fluorescent dyes, SRB and FSS, were used as model drugs. These two dyes were selected due to their good solubility in PBS and their different molecular weights: 558 Da (SRB) and 376 Da (FSS).

**Figure 5:**
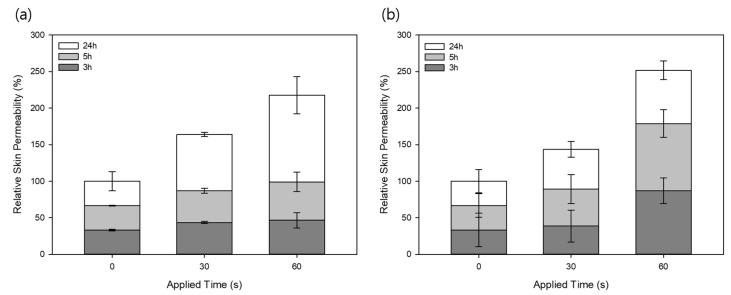
Relative skin permeability of (**a**) sulforhodamine B (SRB) and (**b**) fluorescein sodium salt (FSS) after applying 1200 MLPs for 30 and 60 s (means ± SD, *n* = 3).

When the porcine skin was treated with 1200 MLPs, the relative skin permeability of the SRB to the control group after 3 h was 33.3% ± 0.9%, 43.5% ± 1.4% and 46.7% ± 10.5%, 5 h was 66.7% ± 0.4%, 87.2% ± 3.3% and 99.1% ± 13.1%, and 24 h was 100% ± 13.2%, 163.4% ± 2.9% and 217.6% ± 25.6%, when applied for 0 (control), 30 and 60 s, respectively, shown in Figure 5a. Most drugs used for transdermal delivery have a molecular weight of less than 500 Da, and research has shown that compounds with a molecular weight larger than 500 Da are unable to penetrate the stratum corneum [25,26,27]. Additionally, it has been observed that the amount of time for molecules to penetrate the skin increases as the molecular weight is increased [28]. Accordingly, SRB might have taken longer to pass through the stratum corneum and the lipid matrix layer underneath, and hence, the fluorescent dye might have not fully permeated across the skin. Therefore, no big difference was observed between the three groups after 5 h. However, after 24 h, the relative skin permeability was 163.8% for the 30 s MLP treatment and 217.6% for the 60 s treatment. Even though the SRB has a molecular weight larger than 500 Da, it was clear that the MLPs were able to disrupt the stratum corneum and consequently increase the skin permeability of the drug.

Next, the permeability of FSS, which has a molecular weight smaller than 500 Da, was investigated. Unlike the larger fluorescent dye, the relative permeability of FSS was greater in the MLP treated group than in the control group during all time points. As seen in Figure 5b, the relative permeability of FSS to the control group after 3 h was 33.3% ± 22.9%, 38.9% ± 21.7%, and 87.1% ± 17.4%, 5 h was 66.7% ± 16.1%, 89.3% ± 19.8%, and 178.9% ± 18.9%, and 24 h was 100% ± 16.0%, 143.6% ± 10.9%, and 251.7%± 12.6%, when applied for 0, 30, and 60 s, respectively. Overall, the permeability of the fluorescent dye after 24 h was greater than that of SRB for both the 30 and 60 s groups, indicating that the stratum corneum might not have been as effective of a barrier to fluorescein compared to SRB due to the former’s smaller size and that the MLPs successfully disrupted the stratum corneum for increased skin permeability.

### 3.5. Transdermal Delivery of Niacinamide after MLP Treatment

Niacinamide is a vitamin B3 derivative that acts as a skin whitening agent due to its ability to inhibit the melanosomal transfer from melanocytes to keratinocytes [29,30]. Although it is widely used as a cosmeceutical agent, the hydrophilic characteristic of niacinamide prevents it from being easily absorbed by the skin, despite its small molecular weight of 122 Da. Therefore, the MLPs were tested for their ability to increase the relative skin permeability of niacinamide.

**Figure 6:**
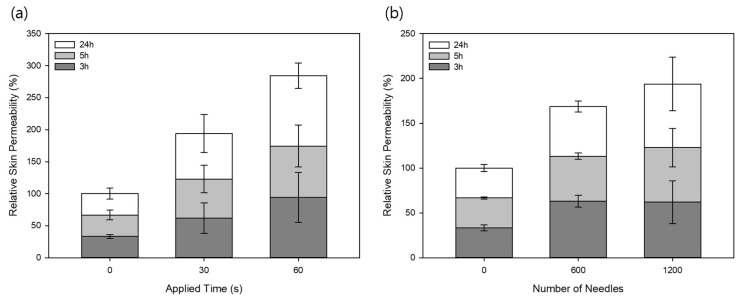
Relative skin permeability of niacinamide after (**a**) applying 1200 MLPs for 30 and 60 s. The same experiment was performed again using niacinamide to determine the relative skin permeability after (**b**) applying 300 and 600 MLPs for 30 s (means ± SD, *n* = 3).

Similarly to the experiment performed using the two fluorescent dyes, porcine was treated with 1200 MLPs for 30 and 60 s. Because niacinamide has a smaller molecular weight than both SRB and FSS, it was expected that the relative skin permeability would be higher. The results, shown in Figure 6a, demonstrate that this hypothesis was proven to be true because a higher relative permeability of niacinamide was observed at all three time points. The relative skin permeability of niacinamide after 3 h was 33.3% ± 3.2%, 62.0% ± 23.9%, and 94.2% ± 39.2%, 5 h was 66.7% ± 7.61%, 122.8% ± 21.6%, and 174.4% ± 32.8%, and 24 h was 100% ± 8.6%, 193.8% ± 29.9%, and 284.3% ± 20.0% when the MLPs were applied for 0, 30 and 60 s, respectively. Another study from Fang et al. observed that the skin permeability of 5-aminolaevulinic acid (ALA), a hydrophilic molecule with a molecular weight similar to niacinamide, after microdermabrasion treatment was about 5-fold higher than that of normal skin [31]. This is not significantly higher than the enhanced permeability obtained using the MLPs, and taking into consideration the fact that microdermabrasion techniques require a separate microabrasor machine and non-dissolving crystals, MLPs do not leave any waste and are more convenient to use.

Apart from the time dependence of the MLPs on enhancing the relative skin permeability of niacinamide, the effect of the number of MLPs was also investigated. After applying the needles onto porcine skin and quantifying the niacinamide using HPLC, the results demonstrate that when 600 MLPs were used, the relative permeability was 63.0% ± 6.7%, 113.1% ± 3.6%, and 168.6% ± 5.9%, whereas the skin treated with 1200 MLPs had a permeability of 62.0% ± 23.9%, 122.8% ± 21.6%, and 193.8% ± 29.9%, and Non-MLP treated group was 33.3% ± 3.2%, 66.7% ± 1.1%, and 100% ± 4.0%, after 3, 5, and 24 h later, respectively, shown in Figure 6b. This is consistent with the results obtained from a different study in which it was discovered that as the number of crystals used for dermabrasion increased, the amount of removed stratum corneum increased as well [32]. Hence, there was a clear indication that as more MLPs were applied, the more they disrupted the stratum corneum layer resulting in an increased permeation of niacinamide. However, the permeability of the group treated with 1200 MLPs was not substantially larger than the group treated with 600 MLPs. As a result of applying the MLPs onto a small area of the porcine skin, there might have been a saturation of the particles. Hence, when the skin was treated with 1200 MLPs, it might not have been as efficient in disrupting the stratum corneum layer as it did with the 600 MLP treatment. Therefore, finding the right amount of MLPs for different surface areas can lead to a more efficient treatment.

### 3.6. Histological Analysis

**Figure 7:**
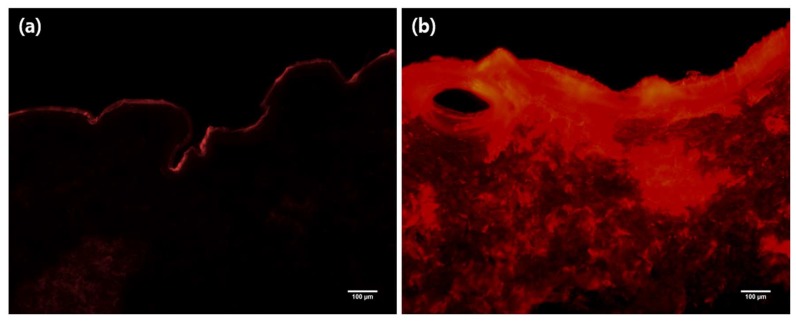
Histological images of cryosectioned porcine skin after SRB dye application. Dye distribution of (**a**) MLP untreated and (**b**) MLP treated porcine skin observed under fluorescent microscope.

To determine the distribution of drugs in skin treated with MLPs, a SRB solution was applied onto porcine skin and cryosectioned into 30 µm slices for observation under a fluorescent microscope. As seen in Figure 7a, when the stratum corneum layer was intact, the SRB solution was not able to penetrate into the deeper layers, and instead, it stayed accumulated in the outermost layer. On the other hand, Figure 7b shows that in the skin treated with the MLPs, the SRB solution was able to permeate through the lower layers of the skin and pass the stratum corneum as a result of its disruption. Thus, it was visually demonstrated that the MLPs were properly designed with the required mechanical strength to disrupt the skin and facilitate the permeation of hydrophilic compounds across the stratum corneum.

## 4. Conclusions

This study demonstrated that the MLPs were capable of disrupting the skin layer to increase the skin permeability of hydrophilic drugs like niacinamide. The results show that the MLPs were capable of being fabricated with reproducibility and that the structure had sufficient mechanical strength to withstand the continuous rubbing of the MLPs to disrupt the skin. Furthermore, the Franz diffusion cell experiment results demonstrated that the permeability of SRB and FSS increased to as much as 217% ± 25.6% and 251% ± 12.8%, respectively, and were dependent on the application time. Additional analysis with niacinamide revealed that when more needles were applied to the skin for a duration of 30 s, the skin permeability increased to about 193.8% ± 29.9%. Fluorescence images of porcine skin slices further show that the MLPs were able to disrupt the skin such that the fluorescent dye was able to penetrate deeper. Overall, the results demonstrated that the MLPs were able to effectively deliver niacinamide to the skin. Future work should focus on investigating the incorporation of the MLPs onto existing formulations. The effect that the ingredients in the formulation have on the MLPs needs to be observed to ensure that the needles are able to maintain its chemical and physical properties. Furthermore, the application of MLPs onto mice must be performed, as it is a closer simulation to the human application.

## Figures and Tables

**Table 1:** Similarities and differences between MLPs and conventional microneedles.

Type	Microneedle	MLP
Similarity	Micrometer Scale	
	Fabricated using biodegradable materials employing already established micromolding techniques	
	Disrupt skin layer to enhance permeability	
Difference	Needles protrude in a single direction	Needles protrude in three different directions
	Applied as a patch	Individual particles rubbed onto the skin
	Applied over a small surface area	Applied over a large surface area
